# Geographically Weighted Regression Modeling of Spatial Clustering and Determinants of Focal Typhoid Fever Incidence

**DOI:** 10.1093/infdis/jiab379

**Published:** 2021-11-23

**Authors:** Venkata Raghava Mohan, Manikandan Srinivasan, Bireshwar Sinha, Ankita Shrivastava, Suman Kanungo, Kulandaipalayam Natarajan Sindhu, Karthikeyan Ramanujam, Santhosh Kumar Ganesan, Arun S Karthikeyan, Senthil Kumar Jaganathan, Annai Gunasekaran, Alok Arya, Ashish Bavdekar, Temsunaro Rongsen-Chandola, Shanta Dutta, Jacob John, Gagandeep Kang

**Affiliations:** 1 Department of Community Health, Christian Medical College, Vellore, India; 2 Wellcome Trust Research Laboratory, Division of Gastrointestinal Sciences, Christian Medical College, Vellore, India; 3 Centre for Health Research and Development–Society for Applied Studies, New Delhi, India; 4 KEM Hospital Research Centre, Pune, India; 5 National Institute of Cholera and Enteric Diseases, Kolkata, India

**Keywords:** typhoid incidence, hotspots, SaTScan clusters, spatial autocorrelation, geographically weighted regression

## Abstract

**Background:**

Typhoid is known to be heterogenous in time and space, with documented spatiotemporal clustering and hotspots associated with environmental factors. This analysis evaluated spatial clustering of typhoid and modeled incidence rates of typhoid from active surveillance at 4 sites with child cohorts in India.

**Methods:**

Among approximately 24 000 children aged 0.5–15 years followed for 2 years, typhoid was confirmed by blood culture in all children with fever >3 days. Local hotspots for incident typhoid cases were assessed using SaTScan spatial cluster detection. Incidence of typhoid was modeled with sociodemographic and water, sanitation, and hygiene–related factors in smaller grids using nonspatial and spatial regression analyses.

**Results:**

Hotspot households for typhoid were identified at Vellore and Kolkata. There were 4 significant SaTScan clusters (*P* < .05) for typhoid in Vellore. Mean incidence of typhoid was 0.004 per child-year with the highest incidence (0.526 per child-year) in Kolkata. Unsafe water and poor sanitation were positively associated with typhoid in Kolkata and Delhi, whereas drinking untreated water was significantly associated in Vellore (*P* = .0342) and Delhi (*P* = .0188).

**Conclusions:**

Despite decades of efforts to improve water and sanitation by the Indian government, environmental factors continue to influence the incidence of typhoid. Hence, administration of the conjugate vaccine may be essential even as efforts to improve water and sanitation continue.

Typhoid fever, caused by *Salmonella enterica* serovar Typhi, is an important public health problem leading to substantial morbidity and mortality. The disease burden varies between and within countries. The World Health Organization estimates that worldwide around 11–20 million people acquire the disease and >150 000 people die annually [1–3].

In India, a recent systematic review estimated prevalence of typhoid fever from various hospital-based studies at 7% (95% confidence interval [CI], 5.7%–16.0%) and was higher during local disease outbreaks. Information regarding the burden of typhoid fever in community settings from India was limited. The pooled incidence of laboratory-confirmed typhoid fever across all studies was 377 (95% CI, 178–801) per 100 000 person-years and highest among children aged 2–4 years, with significant heterogeneity between different studies [4].

Several epidemiological studies have identified either water or foodborne routes as the primary mechanisms involved in transmission of typhoid fever. The disease is higher in population that lack access to safe water and adequate sanitation. Economically poorer communities and vulnerable population subgroups, including children, are at highest risk. Household-level characteristics including larger household size, recent typhoid fever in the household, members not using soap for hand washing, sharing food, and absence of a toilet are documented independent risk factors for typhoid fever [2, 5–7].

Geographic information science has been used to study at-risk areas and spatial clustering of typhoid fever. Typhoid, as with other diarrheal diseases, often exhibits geographic patterns due to its feco-oral mode of transmission, mainly through shared water supplies. Spatial and temporal clustering of typhoid cases has been documented at subnational levels [8–11], with local hotspots within smaller geographic regions associated with environmental factors [12–14]. Studies on typhoid examining spatial patterns and relationships at still finer scale of local neighborhoods and living environments are limited.

The multicentric Surveillance for Enteric Fever in India (SEFI) study was conducted to obtain reliable and high dimensional estimates of burden of typhoid fever using multiple strategies in both urban and rural settings across the country [15]. This paper mapped and evaluated spatial clustering of typhoid fevers; detected hotspots; and modeled incidence rates of typhoid fever, evaluating relationships with family-level sociodemographic and water, sanitation, and hygiene (WASH) practices in the 4 cohorts of the SEFI study.

## METHODS

### Data Sources and Spatial Mapping

The multicentric SEFI study was initiated in October 2017 with the objectives of (1) estimating the incidence of typhoid fever in children between 6 months and 15 years of age across 4 sites—3 urban (Vellore, Kolkata, and Delhi) and 1 rural (in Pune)—though an active community-based cohort surveillance (tier 1); (2) estimating the incidence of severe typhoid fever in all ages using a hybrid approach combining hospitalization and healthcare utilization data; (3) monitoring patterns of antimicrobial use and antimicrobial resistance in patients with typhoid fever; and (4) estimating cost and consequences of typhoid fever in different healthcare settings (tiers 2 and 3) in India [15].

The detailed study protocol, including sample size calculations, has been published previously [15], and the study was registered with the Clinical Trial Registry of India (CTRI/2017/09/009719). Sociodemographic and geospatial data and information on occurrence of blood culture–confirmed typhoid fevers among the study participants were obtained from the active surveillance (tier 1) component of the SEFI study. Geocoordinates of residences of consenting study families were collected during enrollment and subsequent home visits during follow-up of acute febrile illnesses in the community using electronic survey instruments. Spatial mapping of the boundaries of the study area was performed by trained field research assistants using handheld global positioning devices. Study area polygons and study houses were mapped, and spatial analyses were performed using ArcGIS Desktop 10.7.1 software [16].

## Spatial Analysis

### Spatial Clustering and Hotspot Analyses

Local hotspots for incident cases of typhoid fever were assessed using the Optimized Hot Spot Analysis by Getis-Ord Gi* local statistic tool in ArcGIS 10.7 to detect statistically significant spatial clusters of high values (hotspots) and low values (coldspots) across the 4 study sites. The statistic returns a *z* score for each feature in the dataset; the larger the *z* score, the more intense the clustering of high values (hotspots) [17–22].

SaTScan software version 9.7, developed by Kulldorff, was used to detect and evaluate spatial clusters of typhoid fever at the sites using a purely spatial Poisson-based scanning model running a circular scanning window. SaTScan scans across time and/or space were used to identify possible clusters by comparing the number of observed events and expected events inside the window at each location. Clusters with significant levels with cutoff values such as .05, .01, and .001 after repeated Monte Carlo simulations were reported. The cluster with the maximum log likelihood ratio was taken as the most likely cluster (least likely to be due to chance). Secondary clusters in the regions were also identified, and only nonoverlapping significant spatial clusters with high rates are presented [11, 23–28].

### Incidence of Typhoid Fever and Spatial Autocorrelation Analysis

Since the rural cohort in Vadu, Pune had sparse cases of typhoid fever during the entire study duration, this site was excluded from further spatial analyses.

Polygon feature classes of tessellated regular hexagonal grids were created for each of the study sites with an approximately 2500 m^2^ area (50 m × 50 m) using geoprocessing tools in ArcGIS 10.7 [29, 30]. Incident cases of typhoid were aggregated for these grids and incidence rates for the study duration were estimated for the grids. The zero inflated incidence data was normalized using the double arcsine Freeman–Tukey transformation technique [31–33]. The formula for the Freeman–Tukey double-arcsine transformation was


$$f(r,n)=\arcsin\left(\sqrt{\frac{r}{n+1}}\right)+\arcsin\left(\sqrt{\frac{r+1}{n+1}}\right)$$

where *r* = number of events and *n* = person-time of follow-up.

To assess the degree of clustering and spatial patterns at each of the 3 sites (Vellore, Kolkata, and Delhi), local spatial autocorrelation was assessed using local indicators of spatial association, estimated using the Anselin local Moran *I* statistic for transformed typhoid incidence rates (per child-year [CY]) in the hexagonal grids using the Cluster and Outlier analysis tool in ArcGIS 10.7. A positive value for the statistic I indicated that a grid had neighboring grids with similar high or low incidence rates and was a part of a cluster. A negative value for *I* indicated that the grid had neighbors with dissimilar values. Spatial autocorrelation classified the features into “hotspots” (high values surrounded by high [HH]) or “coldspots” (low values next to low [LL]) and outlier clusters such as HL (high among low neighbors) or LL (low among low neighbors); those grids with *P* values <.05 were considered statistically significant [21, 34–38].

### Spatial Regression Analysis

To better understand the influence of sociodemographic and WASH-related factors behind observed disease incidence rates in the local tessellated grids, initially, a global regression method—the ordinary least squares regression (OLS) analysis [39, 40]—was performed individually across 3 sites (Vellore, Kolkata, and Delhi) using the spatial statistics tools in ArcGIS 10.7. The number of study families, proportions of families in the lower socioeconomic strata, overcrowding, unsafe water and sanitation, untreated drinking water, purchasing ready-to-use food from local small vendors, and children eating locally sold ice candy from street vendors in the grids were included as explanatory variables while the transformed typhoid incidence rates at the hexagonal grid was the outcome variable. Variation inflation factors, corrected Akaike information criterion (AICc) estimates, and *R*^2^ values that explain the variability in the dependent variable were considered to determine the best-fitting model at the sites [39].

Spatial autocorrelation of variables and spatial variations (nonstationary nature) of explanatory variables pose a challenge in meeting the requirements of a nonspatial statistical analysis like the OLS regression. Geographically weighted regression (GWR) is a local regression analysis that can examine relationships at every feature level (grids in this case) and calibrate using nearby features assuming spatial nonstationarity, indicating that the correlations between the outcome and predictor variables are not the same for every feature [38, 41–45]. A grid-level GWR was performed using the explanatory and outcome variables as in the OLS regression. Coefficients for different predictor variables were assessed and the standard residuals from the model were mapped for the 3 sites. The larger the coefficient of a predictor variable, the stronger is its relationship to the incidence of typhoid fever. Over/underestimation of typhoid incidence in the grids was assessed and spatial autocorrelation of the standard residuals was performed using the global Moran *I* statistical tool to examine whether the obtained patterns were either clustered, random, or dispersed. While the *z* scores and *P* values indicate statistical significance, a positive Moran *I* index value indicates tendency toward clustering and a negative value indicates tendency toward dispersion [46, 47].

## RESULTS

**Figure 1.**
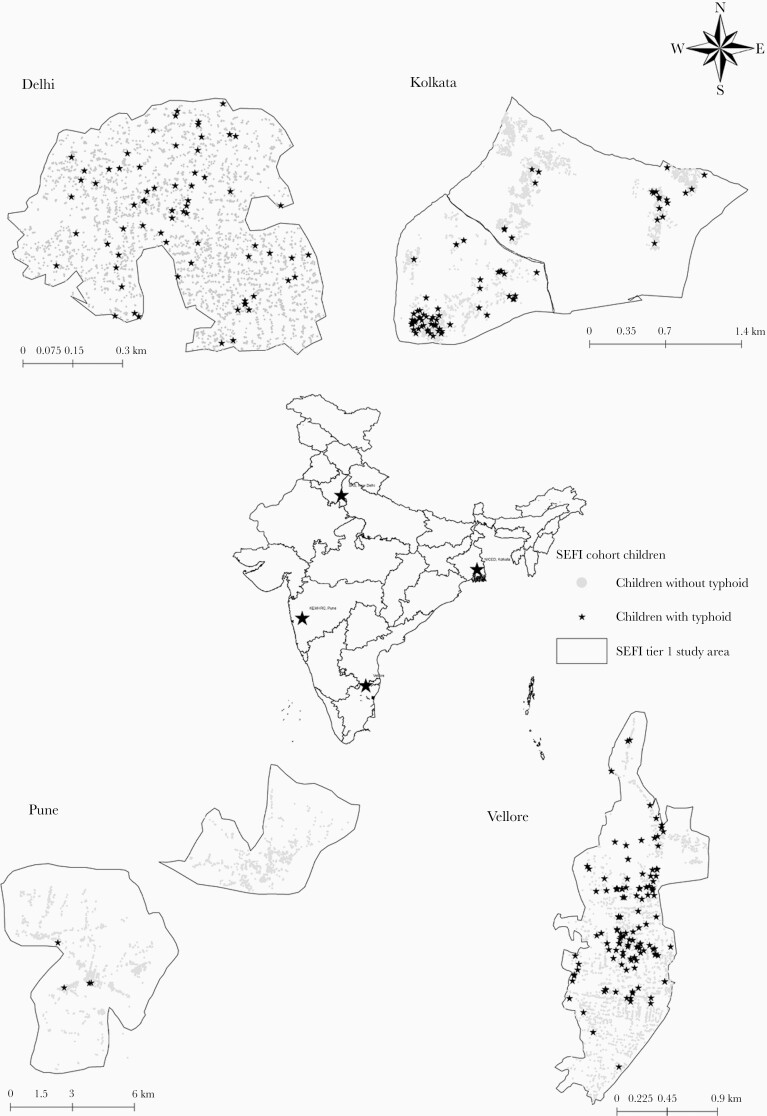
Location of the 4 cohort sites in the Surveillance for Enteric Fever in India (SEFI) study. The 3 urban sites included Vellore, Kolkata, and Delhi. The only rural site was in Vadu, Pune.

A total of 24 062 children were enrolled across 4 sites, with 21 470 (89.2%) completing 24 months of follow-up. Of the 4 sites, Vellore, Kolkata, and Delhi represented urban communities with geographic areas of 2.20, 4.15, and 0.42 km^2^, respectively, while the site in Vadu, Pune with an area of 79 km^2^ represented the only rural community in the tier 1 component of the SEFI study. During the study period, 299 incident cases of blood culture–confirmed typhoid fever were documented across the 4 sites; the highest number of cases was 146 from Vellore and there were only 4 cases from Vadu, Pune. Cases of typhoid fever were distributed across the study sites except in Vadu, where all 4 cases were observed in 1 locality (Figure 1).

### Spatiotemporal Distribution of Typhoid Fevers

**Figure 2.**
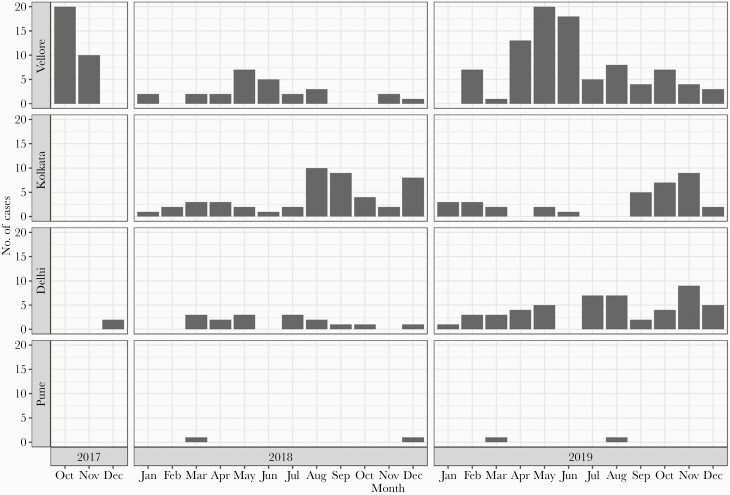
Seasonality in occurrence of typhoid cases across the 4 Surveillance for Enteric Fever in India (SEFI) cohort sites. This graph presents the occurrence of incident typhoid cases during different months across the 4 cohorts. Enrollment in Vellore started in late 2017, whereas the other sites started in 2018.

Of the 299 typhoid cases, 30 (10%) were documented during the last quarter of 2017 from Vellore. A total of 91 and 176 cases were recorded from all 4 sites for the years 2018 and 2019, respectively. Cases of typhoid occurred in almost all months in Vellore and Kolkata. In Vellore, peak cases (56%) occurred during the second quarter (April to June) of 2018 and 2019, while 72% (58/81) and 64% (42/66) of cases were in the last 2 quarters in Kolkata and Delhi, respectively. Cases were densely distributed across 2 geographical localities in Vellore and Kolkata during the peak seasons, but no specific spatial pattern was seen in the denser Delhi cohort. Seasonality of incident typhoid cases and their spatial distribution are presented in Figure 2 and Supplementary Figures 1 and 2.

### Spatial Clustering and Hotspots of Typhoid Fever

**Figure 3.**
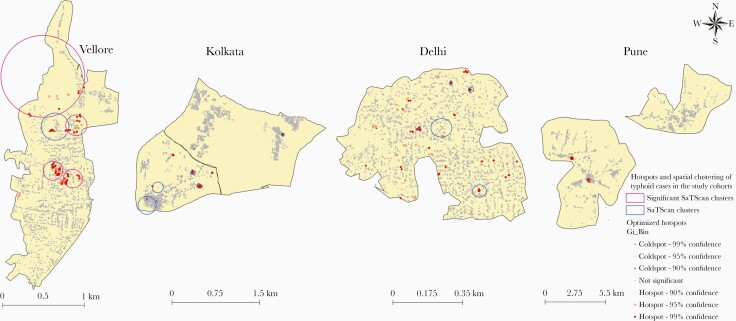
Spatial hotspots and clustering of typhoid cases in the Surveillance for Enteric Fever in India (SEFI) study cohorts. Hotspot households for typhoid were present in all 4 sites, whereas significant SaTScan clustering was detected only in Vellore.

Aggregating spatial data on incident cases of typhoid fevers and correcting for both multiple testing and spatial dependence, a total of 856 cohort households were identified as significant hotspots for typhoid at the 4 sites using the optimized Getis-Ord Gi* statistic. Vellore with 146 typhoid cases had 365 hotspot cohort households whereas Kolkata, Delhi, and Pune had 131, 175, and 186 households identified as potential hotspots during the study period (Figure 3).

A purely spatial SaTScan scanning for clusters with high incident cases of typhoid fever using a discrete Poisson model revealed 4 statistically significant clusters (*P *< .05) for typhoid fever in urban communities of Vellore with the relative risks and radii being 5.4 and 0.5 km; 5.0 and 0.11 km; 4.0 and 0.13 km; and 3.6 and 0.12 km, respectively. Even though 2 spatial clusters were detected in Kolkata and Delhi sites, these were not statistically significant (Figure 3).

### Focal Incidence of Typhoid Fever in the Study Cohorts

**Figure 4.**
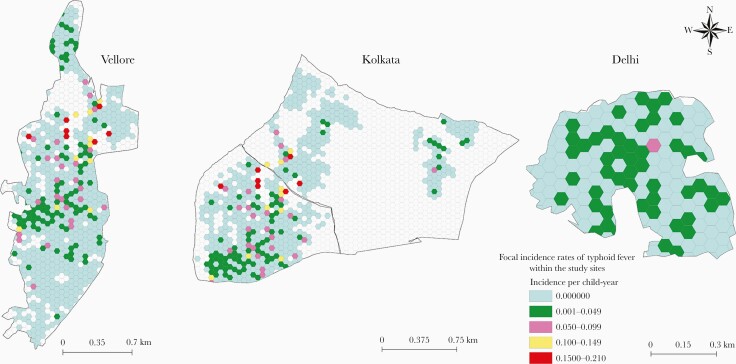
Focal incidence of typhoid fever across 3 Surveillance for Enteric Fever in India cohort study locations. Local neighborhoods with high typhoid incidence rates were present in Vellore and Kolkata and appeared to be randomly distributed.

There were 2421 hexagonal grids with at least 1 study household across the 4 study sites. The mean numbers of people and children aged <15 years per grid were 30.1 (standard deviation [SD], 0.4) and 11.7 (SD, 16.9) respectively. Mean incidence of typhoid fever in the grids was 0.004 (SD, 0.022) per CY and the highest incidence was 0.526 per CY. The Vellore and Kolkata sites had a few pockets with incidence rates >0.15 per CY (Figure 4).

### Local Spatial Autocorrelation Analysis

**Figure 5.**
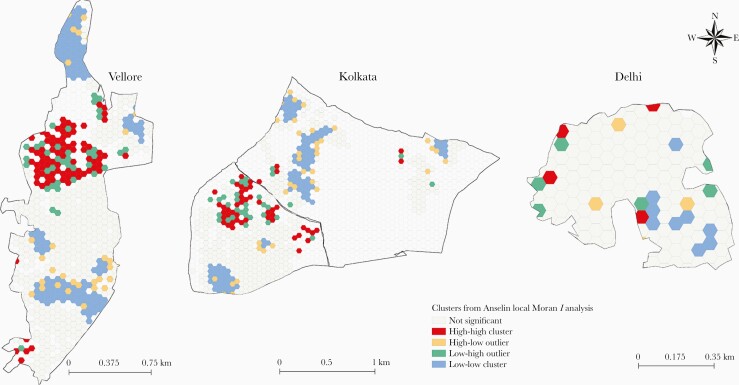
Spatial patterns and clustering of typhoid disease in neighborhoods across 3 Surveillance for Enteric Fever in India study cohort sites. High-high clusters for typhoid disease incidence were detected in Vellore and Kolkata and demonstrated specific spatial patterns.

Of the 1503 tessellated grids with study households across Vellore, Kolkata, and Delhi, 132 (8.7%) were high-high (HH) clusters for typhoid fever. Vellore had 11.4% (80/704) HH clusters concentrated in 2 local neighborhoods toward the middle and south of the study area. In Kolkata, the HH clusters (48/624 [7.7%]) were located toward the western part of the study area; the Delhi site, with only 2.3% of grids being HH clusters, did not demonstrate any geographic pattern (Figure 5).

### Sociodemographic Predictors of Typhoid Fever Incidence

Exploratory regression analysis using the multivariate OLS regression to model incidence of typhoid and its relationship to collected family-level sociodemographic and WASH practices at the grid level revealed that not all variables were consistently associated with burden of typhoid across the 3 locations.

The number of study families in the grids consistently showed a significantly negative association across all 3 locations, while the proportion of overcrowded families was positively associated with disease incidence in the Delhi site. Unsafe water and poor sanitation were positively associated, although not statistically significant, in Kolkata and Delhi. The proportion of families drinking untreated drinking water was significantly associated with typhoid incidence in both the Vellore (*P *= .034) and Delhi (*P *= .018) cohorts whereas in Kolkata, a nonsignificant positive association was detected. The proportion of families buying ready-to-use cooked food from local vendors associated with typhoid, was significantly higher in Kolkata (*P *= .0004). The proportion of variance (adjusted *R*^2^) in the incidence of typhoid fever in these cohorts as explained by the modeled predictor variables ranged from 15% to 21.7%. A summary of the multivariate OLS with model diagnostics is provided in Table 1.

**Table 1.** Multivariate Ordinary Least Squares Regression and Geographic Weighted Regression Models Using Transformed Incidence of Typhoid Fever at the Vellore, Kolkata, and Delhi Sites of the Surveillance for Enteric Fever in India Study

Variable	Best-Fitting OLS Models at 3 Sites								
	Vellore			Kolkata			Delhi		
	β	SE	*P* Value	β	SE	*P* Value	β	SE	*P* Value
Intercept	.377277	0.029752	<.0001^a^	.366093	0.039275	<.0001^a^	.171992	0.080425	.0339^a^
No. of study families in the grid	–.006046	0.000557	<.0001^a^	–.00685	0.000644	<.0001^a^	–.00441	0.00077	<.0001^a^
Proportion of families belonging to lower socioeconomic status	–.010989	0.020173	.5861	–.01277	0.024091	.5963	.058593	0.050533	.2479
Proportion of overcrowded families	–.013031	0.026886	.6280	–.0019	0.037766	.9599	.033664	0.073906	.6493
Proportion of families with access to unsafe water	–.033016	0.07819	.6729	.023812	0.042469	.5752	.073252	0.218459	.7378
Proportion of families not treating drinking water before use	.036626	0.017264	.0342^a^	.013631	0.024785	.5825	.081211	0.03423	.0188^a^
Proportion of families with unsafe sanitation	–.019394	0.017466	.2672	.026532	0.025532	.2991	.020172	0.087223	.8173
Proportion of families buying ready-to-use food from local shops	–.011993	0.01705	.4820	.081287	0.022987	.0004^a^	.039254	0.044708	.3812
Proportion of families with children consuming locally sold ice candy	.034116	0.02349	.1468	.033133	0.026364	.2093	.044577	0.033017	.1788
AICc	–615.08			–396.05			–266.79		
Adjusted *R*^2^	0.157			0.187			.218		
AICc (GWR)	–726.94			–445.95			–268.62		
Adjusted *R*^2^ (GWR)	0.323			0.271			.226		

Abbreviations: AICc, corrected Akaike information criterion; GWR, geographic weighted regression; OWS, ordinary least squares; SE, standard error.

^a^P < .05 is considered statistically significant.

A locally linear, nonparametric estimation using the GWR was used to deal with the nonstationary nature of the OLS regression. Even though GWR did not improve the model fits from the OLS model according to the AICc values, the adjusted *R*^2^ values increased from 0.15 to 0.32; 0.18 to 0.27; and 0.21 to 0.22 across the 3 cohorts (Table 1).

**Figure 6.**
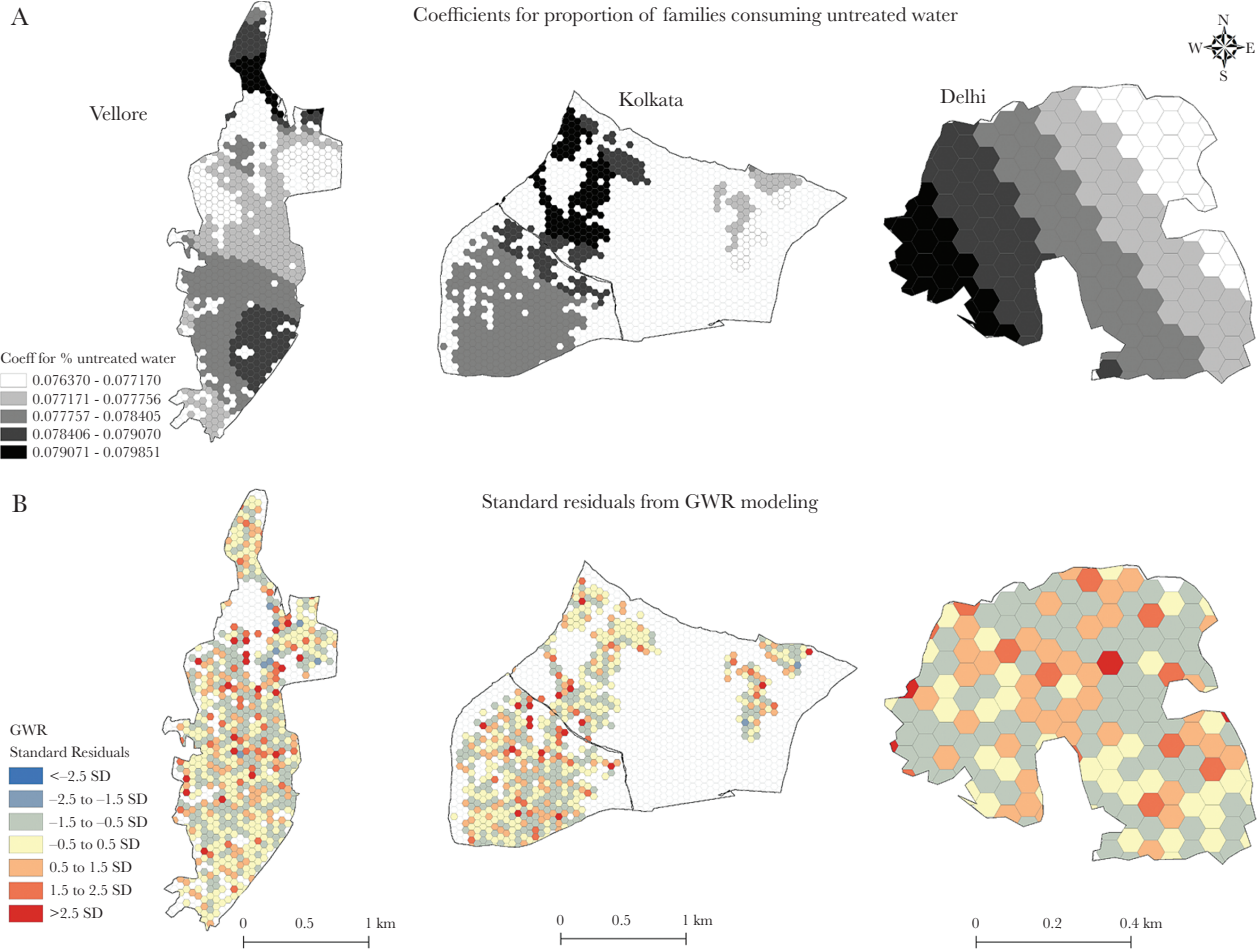
Mapping coefficients (*A*) and standard residuals (*B*) of the geographically weighted regression analysis. Coefficients for proportion of families consuming untreated water exhibited distinct spatial patterns, and there was no overestimation with a randomly distributed underestimation of typhoid incidence in the grids. Abbreviations: GWR, geographically weighted regression; SD, standard deviation.

The mapped coefficients for proportion of families drinking untreated water and standard residuals of the GWR across 3 cohort locations are presented in Figure 6.

The proportion of families consuming untreated water in the hexagonal grids was a strong predictor in the northern and southeastern parts of the Vellore catchment area, whereas it was more in the centrally distributed families in Kolkata and in the western neighborhood in the Delhi catchment area (Figure 6A). Mapping of standard residuals of the GWR revealed absence of overestimation (blue grids) and a random distribution of underestimation (red grids) of typhoid incidence across the 3 regions as indicated by the Moran indices for standard residuals (Figure 6B). Moran *I* values for Vellore, Kolkata, and Delhi were 0.0121 (*P *= .51), 0.0161 (*P *= .28), and –0.0676 (*P *= .26), respectively.

## Discussion

The 4 locations included in the study varied in terms of catchment area, with the urban area in Delhi spanning 0.42 km^2^ and the only rural site in Pune distributed over 79 km^2^. Typhoid disease incidence rates varied across the sites: lowest in the rural location (35 per 100 000 CY) and the highest in periurban communities of Vellore (1173 per 100 000 CY) (John et al, unpublished data), indicating wide heterogeneity in disease burden among children in the study cohorts.

In Vellore, typhoid cases were documented consistently during the study period except during the months of May–July in the year 2019 when the highest number of cases occurred coinciding with the relaying of a sewage network in these urban neighborhoods as part of the Smart City campaign, which could have resulted in a local outbreak of typhoid fever. In Kolkata and Delhi, higher numbers of cases were seen during the last 2 quarters, which normally are the monsoon and postmonsoon seasons. During the peaks, cases of typhoid were spatially located in certain localities of the study areas in Vellore and Kolkata, indicating limited geographic spread in these cohorts. In Pune, of the 4 cases, 2 were in the same locality but during different time points.

In Vellore, houses within close proximity (within a radius of 150 m) of a typhoid case were at significantly higher risk of typhoid, indicating pockets of focal transmission around the case houses, likely due to environmental exposure to contaminated water or food. Although similar hotspot households were detected in the Kolkata study site, there was no significant spatial clustering of cases. Since spatiotemporal analyses was not performed, we could not assess whether these hotspots or clusters varied with both time and space at the study sites.

Since incident typhoid cases demonstrated specific spatial patterns in these cohorts, disease burden and relationship with predictors were assessed at grid level. Three pockets with highest typhoid incidence (>0.25 per CY) were detected at the Kolkata site while 1 pocket each in Vellore and Delhi had incidence rates of 0.21 and 0.08 per CY, respectively. The locations of significant clusters with higher typhoid incidence (HH clusters) did not spatially overlap with the clusters of typhoid cases as anticipated since incidence was estimated for all the households within the grid at grid level and is a function of person-time of follow-up.

Studies have documented that in addition to sociodemographic factors and WASH-related behaviors, typhoid occurrence is influenced by neighboring regions [7, 14, 48–50].

Modeling grid level–transformed typhoid incidence with sociodemographic and WASH predictors in this analysis by OLS regression found a negative relationship between number of study families and disease burden at the grid level, which was contrary to expectations. Similarly, we did not detect a positive relationship between proportion of lower socioeconomic status families and disease in both Vellore and Kolkata whereas in Delhi, a nonsignificant positive relationship was seen. Higher population densities and a homogenous distribution of families with similar sociodemographic characteristics across these urban settings may have influenced our findings. The proportion of families that did not practice point-of-use water disinfection was strongly associated with disease burden in the microenvironments studied. These findings suggest that the prevalent community-level water treatment practices were inadequate and do not offer sufficient protection against gastrointestinal diseases. At one of the sites, the practice of purchasing ready-to-use foods by the families was positively associated with typhoid, highlighting the importance of better food hygiene and WASH practices, not only in the families but also in the neighborhoods.

Spatial modeling using the GWR adjusting for immediate neighborhoods and spatial nonstationarity confirmed that there was no overestimation of disease burden at the local grids and any underestimation was a random process in addition to strengthening the findings from the nonspatial regression analysis. Not accounting for meteorological predictors of typhoid disease is one of the limitations in this study.

## Conclusions

The burden of typhoid disease was heterogeneous between urban and rural cohorts in the SEFI study. Local spatial clusters and hotspots among households were present within the urban cohorts. Within each urban cohort, marked variations were noted in the disease incidence rates at local neighborhood resolutions; presence of cases of typhoid in close proximities rendered the group of neighboring households at higher risk of acquiring the disease. Distribution of typhoid risk factors was uneven across the study cohorts. Not practicing point-of-use water treatment and consuming ready-to-use food available in the neighborhoods were significantly associated with disease burden. Despite decades of efforts to improve water and sanitation by the Indian government, environmental factors continue to influence the incidence of typhoid. Hence, administration of the conjugate vaccine may be essential even as efforts to improve water and sanitation continue.

## Supplementary Data

Supplementary materials are available at *The Journal of Infectious Diseases* online. Consisting of data provided by the authors to benefit the reader, the posted materials are not copyedited and are the sole responsibility of the authors, so questions or comments should be addressed to the corresponding author.

**Supplementary Figure 1.** Spatial distribution of incident typhoid cases for the year 2018. 

In the year 2018, higher numbers of typhoid cases were noted in the second quarter in Vellore and in the second and third quarters in Kolkata.

**Supplementary Figure 2.** Spatial distribution of incident typhoid cases for the year 2019. In the year 2019, higher numbers of typhoid cases were noted in the second quarter in Vellore and in the third and fourth quarters in the Kolkata and Delhi cohorts.

jiab379_suppl_Supplementary_Figure_1

jiab379_suppl_Supplementary_Figure_2

## Notes

***Author contributions.*** J. J. and G. K. conceived and designed the study. V. R. M., J. J., G. K., A. B., T. R. C., S. K., B. S., K. N. S., A. S., A. S. K., S. D., and K. R. developed the study protocol and analysis plan. M. S., K. N. S., A. B., A. S., T. R. C., B. S., S. K., S. D., S. K. N., S. K. G., A. G., and A. A. coordinated data collection at the sites. V. R. M., K. R., and S. K. G. analyzed the data. M. S. and V. R. drafted the manuscript. All authors have reviewed the manuscript and approved it. V. R. M., J. J., and G. K. had complete access to data and guarantee the manuscript.

***Acknowledgments.*** We acknowledge with gratitude, the participants, and their families for their participation in the 2 years of surveillance. In addition to members of the Surveillance for Enteric Fever in India (SEFI) consortium, we acknowledge all of the study field personnel for their tireless efforts in gathering reliable data. We acknowledge Nathan Lo and Yanjia Cao for their valuable inputs. We are grateful to Supriya Kumar, Duncan Steele, and Anita Zaidi of the Bill & Melinda Gates Foundation, who have been steadfast in their support of the study with their constructive comments and encouragement. Our gratitude is also due to Soumya Swaminathan, former Secretary of the Department of Health Research, government of India, for providing leadership and participation from the government in the design and conduct of the surveillance.

***Disclaimer.*** The funders had no role in the design, conduct, analysis, interpretation of data, or decision to submit the manuscript for publication.

***Supplement sponsorship.*** This supplement is sponsored by the Christian Medical College Vellore Association.

***Financial support.*** This study was supported by the Bill & Melinda Gates Foundation (grant number INV-009497-OPP1159351) and by the Fogarty International Center, National Institutes of Health (grant number D43 TW007392).

***Potential conflicts of interest.*** All authors: No reported conflicts of interest.
