# Supplementary figures and images for: Geographically Weighted Regression Modeling of Spatial Clustering and Determinants of Focal Typhoid Fever Incidence
Source: J Infect Dis. 2021 Nov 23;224(Suppl 5):S601–11. doi: 10.1093/infdis/jiab379 (PMC8892548; doi:10.1093/infdis/jiab379)

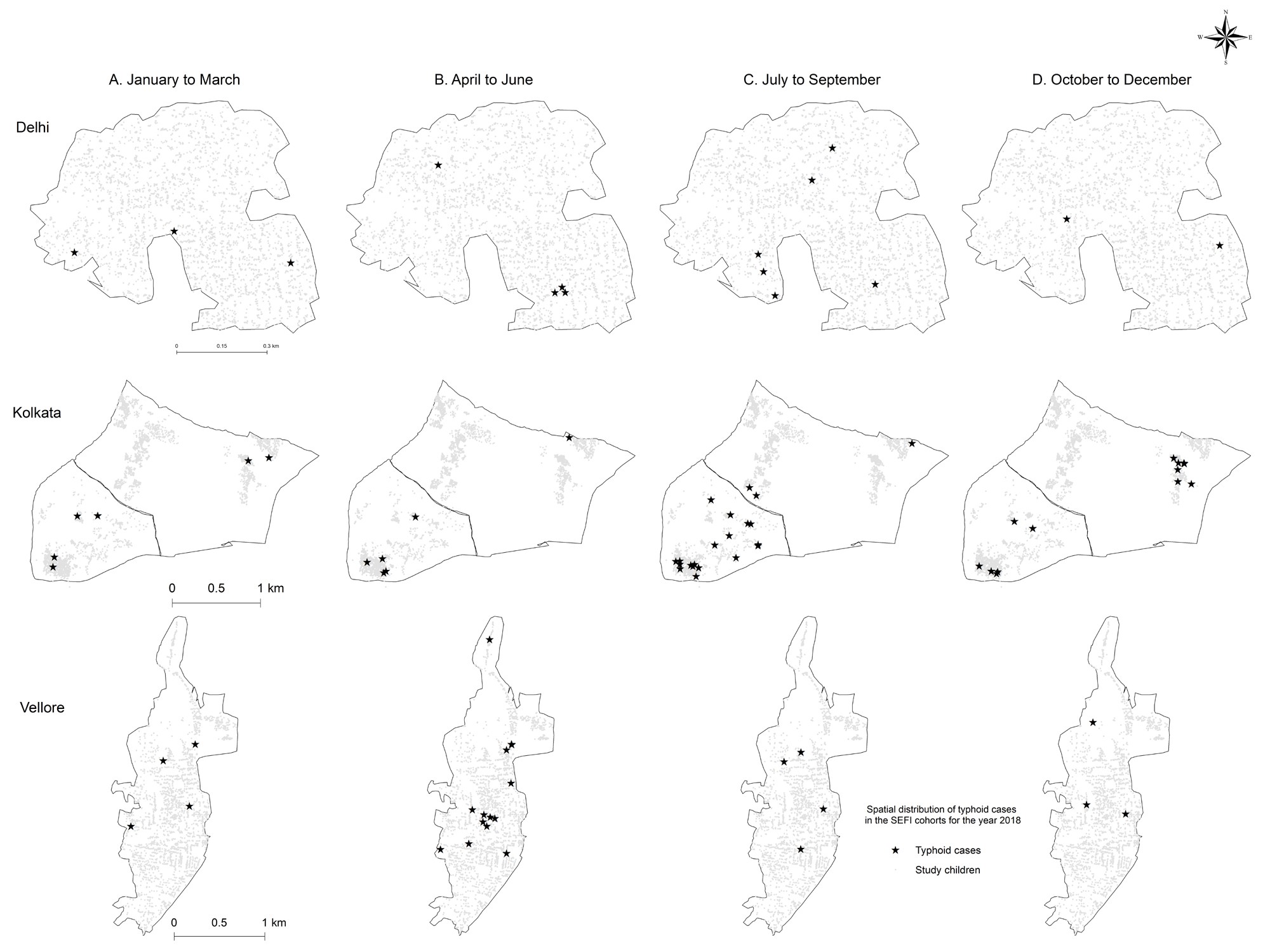

Supplement: jiab379_suppl_Supplementary_Figure_1 [file jiab379_suppl_supplementary_figure_1.jpeg]

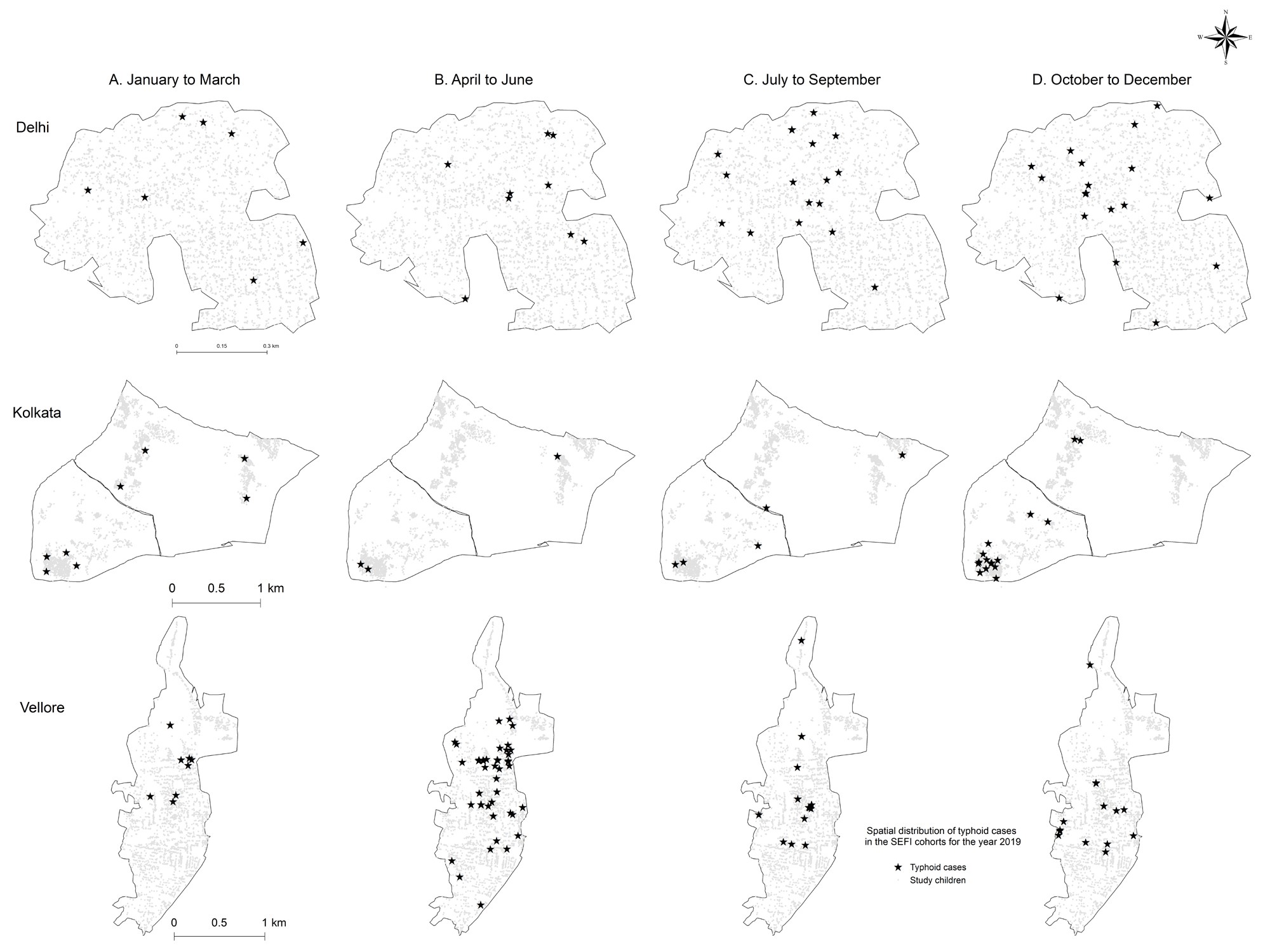

Supplement: jiab379_suppl_Supplementary_Figure_2 [file jiab379_suppl_supplementary_figure_2.jpeg]
